# A “names-as-fixed-effect fallacy” in studies of name-based racial discrimination

**DOI:** 10.1073/pnas.2209603119

**Published:** 2022-08-16

**Authors:** Holger Mitterer

**Affiliations:** ^a^Department of Cognitive Science, University of Malta, Msida MSD 2080, Malta;; ^b^Hanyang Institute for Phonetics and Cognitive Sciences of Language, Hanyang University, Seoul 133-791, Korea

Block et al. (1) performed a large-scale study with more than 250,000 participants who received emails from senders with names associated with a Black or White racial identity. Their main result was that senders with a Black racial identity were less likely to be answered. Given the societal relevance of such results, it is important to make sure they are statistically sound. Given the impressive sample, this might be taken as a given, but the large sample of participants is counteracted by the small sample of only 10 names. In the field of psycholinguistics, it has long been recognized that studies with a sample of linguistic stimuli such as names require tests of whether the results are robust with regard to both participant and item variation (2). More recently, this issue has been also been raised within social psychology (3, 4). In their supplementary materials, Block et al. (1) try to address this issue by redoing their analysis with each of the names being left out, but a better way to test this are generalized linear mixed-effects models that take item variability into account (3). Whereas the original data analysis reported a highly significant effect (*P* < 0.0001), generalized mixed-effects models with a random effect for name indicates that the effect is not robust, neither with the data from the general public (b = −0.108 [log odd units], z = −1.712, *P* = 0.087) nor with the data from elected officials (b = −0.065, z = −1.432, *P* = 0.155).* While this does not mean that we should accept the null hypothesis, it shows that the study is underpowered. This may come as a surprise given the large sample, but statistical power in designs with crossed random effects is strongly constrained by the smaller sample size (i.e., here the sample of only 10 names; see figure 4 in ref. 4).

Block et al. (1) also present a meta-analysis of similar studies; however, none of them took item variation into account in their data analyses and some only use one name per condition. The data from Block et al. (1) show that this is problematic, since the SD in response rate to names within each implied racial identity (0.09 logit units) is about as large as the mean difference between the two groups of names (0.108 logit units). This indicates that there are other effects caused by different names unrelated to implied racial identity. The choice of names in such studies then represents a highly influential researchers’ degree of freedom (5). Since a positive outcome may be much more likely to be published (6) and cited (7), this creates an incentive to use names “that work.” For reasons of statistical power and generalizability, such studies should therefore rely on a large sample of names rather than just five names or even only one name for each treatment level.

## Footnotes

^*^The analyses files can be found here: https://osf.io/swqkf.
